# Family Environment Factors Associated with Symptom Distress Among Korean Adolescents and Young Adults with Cancer: A Cross-Sectional Study

**DOI:** 10.3390/curroncol33070385

**Published:** 2026-06-25

**Authors:** Heeyeon Son, Springer Cary, Sungsil Hong, Jung Woo Han, Cecile Lengacher, Sharron L. Docherty

**Affiliations:** 1 College of Nursing, University of Tennessee, 1200 Volunteer Blvd, Knoxville, TN 37996, USA; 2 Research Computing Support, University of Tennessee, Knoxville, TN 37996, USA; springer@utk.edu; 3 College of Nursing, The Catholic University of Korea, 222 Banpo-daero, Seocho-gu, Seoul 06591, Republic of Korea; hss9456@gmail.com; 4 Department of Pediatric Hematology-Oncology, Yonsei Cancer Center, Yonsei University Health System, 50-1 Yonsei-ro, Seodaemun-gu, Seoul 03722, Republic of Korea; jwhan@yuhs.ac; 5 Department of Pediatrics, Yonsei University College of Medicine, Yonsei University Health System, 50-1 Yonsei-ro, Seodaemun-gu, Seoul 03722, Republic of Korea; 6 College of Nursing, University of South Florida, 12901 Bruce B. Downs Blvd, MDC Box 22, Tampa, FL 33612, USA; cecilel@usf.edu; 7 School of Nursing, Duke University, 307 Trent Dr, Durham, NC 27710, USA; sharron.docherty@duke.edu

**Keywords:** Korean, AYAs, family strength, family support, symptom distress

## Abstract

**Background/objectives**: To describe and compare Korean AYAs’ and parental perspectives on the family environment in terms of agreement and significant differences and examine which variables were associated with AYAs’ symptom distress. **Sample and setting**: Self-report data were collected from a total sample of 113 AYAs, recruited from a pediatric-oncology outpatient clinic at a university-affiliated hospital and community group in South Korea. Because each study aim required different data sources, different analytic samples were used. Specifically, 54 AYA–parent dyads were included for Aim 1, whereas self-report data from 111 AYAs with complete data were used for Aim 2. **Methods and variables**: This subgroup analysis used a quantitative–descriptive, cross-sectional design. AYAs’ and parent perceptions of the family environment (family cohesion and adaptability, family strength, and social support from family) and AYAs’ symptom distress were collected using reliable and validated self-report questionnaires and analyzed using descriptive and inferential statistics. **Results:** AYAs and their parents showed low (family support) to moderate agreement (family strength, family cohesion, and adaptability) on perceptions of family environment (ICC = 0.374–0.612). AYAs reported significantly lower perceptions of family support than their parents, with a small to moderate effect (*p* < 0.001, d = 0.48). All family environment variables were correlated with AYAs’ symptom distress (*p* < 0.05). Among these variables, AYAs’ perceived family strength emerged as the only family environment variable significantly associated with their symptom distress (F = 14.309, *p* < 0.001, R^2^ = 0.359, R^2^adj = 0.334), which was stronger during treatment. **Conclusions:** AYAs’ perceived family strength should be routinely assessed, especially during cancer treatment. Additional nursing interventions focusing on enhancing AYAs’ families as a support group are needed.

## Simple Summary

Cancer-related symptom distress is common in adolescents and young adults (AYAs) and can affect both physical and emotional well-being, but it is often overlooked. Because family support plays an important role in how AYAs cope with cancer, this study explored which aspects of the family environment are linked to lower symptom distress. It also examined whether AYAs and their parents view the family environment in the same way, and whose perspectives were more strongly associated with AYAs’ symptom distress. Using existing data from Korean AYAs with cancer and their parents, the study found that AYAs and parents did not always agree, especially about family support. AYAs also rated family support lower than parents did. Among all family factors, only AYAs’ own perception of family strength was associated with lower symptom distress, especially during treatment. These findings suggest that researchers should pay closer attention to AYAs’ own views when developing family-based supportive care interventions.

## 1. Introduction

Symptom distress is a pervasive and often underrecognized burden among adolescents and young adults affected by cancer (15–39 years, AYAs), with profound consequences for their physical function, psychological well-being, and long-term survivorship [1,2,3]. Symptom distress is a multidimensional construct reflecting the degree of physical and psychological distress experienced in response to cancer- and treatment-related symptoms [4]. According to a recent report, a significantly greater number of AYAs reported moderate to severe symptoms related to pain, tiredness, nausea, depression, anxiety, and poor well-being compared to older adults affected by cancer [5]. The associated financial toll cannot be ignored. According to Korean National Health Insurance data, substantial medical utilization occurred among cancer patients, with an average of USD 5962 in medical expenses during the first year after diagnosis, approximately KRW 7.94 million [6]. Unmanaged symptom distress during adolescence and young adulthood, a critical developmental period, can impose substantial burdens by creating significant barriers to educational attainment and employment among AYAs [2,7]. In this context, there is an urgent need to identify modifiable factors that may alleviate this burden. Despite growing emphasis on managing symptoms and related distress, alleviating physical and psychological symptoms among AYAs affected by cancer remains a major concern in care delivery [2,8].

Symptom distress remains one of the most challenging aspects of the cancer experience for AYAs, yet the role of the family environment in shaping this distress has received comparatively limited empirical attention. The family holds a uniquely vital role in influencing health at both individual and community levels by providing the immediate context that shapes how people develop and maintain their health, adopt health-related behaviors, and receive care and support during illnesses [9]. A growing body of empirical evidence suggests that specific dimensions of the family environment are important correlates of distress-related outcomes among children, adolescents, and AYAs affected by cancer, highlighting the critical role of the family environment [10,11,12,13]. In particular, prior studies have linked family functioning [10,11], family support [12], and parent–child relationships [11] with psychological distress, adjustment, and quality of life in the pediatric and AYA cancer population. For example, prior research has shown that family functioning, which involves family communication, problem solving, and coordination of everyday activities, is associated with AYA survivors’ coping strategies [10]. AYAs who report poorer family functioning are more likely to rely on avoidant coping strategies, which have been linked to worse psychological outcomes. Furthermore, the association between supportive family functioning and improved physical symptom outcomes, including reduced pain, has also been demonstrated in older adults with breast cancer [14]. Together, these findings provide a strong conceptual basis for examining the family environment as a contributor to multidimensional symptom distress, encompassing both physical and psychosocial symptoms among AYAs affected by cancer. In this study, the term “family environment” is used to refer to the broader familial context, with family functioning—including cohesion, adaptability, and shared competence—serving as the primary operational indicators of this environment [15].

Building on this, existing studies are limited in their ability to capture the distinct perspectives of AYAs and their parents regarding the family environment, as many examined the perspectives of one party or combined reports from both groups, thereby obscuring potential differences in perceptions [16]. Nevertheless, evidence suggests that AYAs and their parents often hold divergent views of their family environment [17,18], highlighting the need for analytic approaches that preserve and directly compare informant-specific perspectives.

Several studies have employed dyadic approaches to examine interrelations between AYAs affected by cancer and family members, particularly in relation to perceptions of the family environment and psychological outcomes. For example, Gutiérrez-Colina [19] and Vola et al. [20] investigated associations between survivor–family member reports of the family environment and depressive symptoms or psychological distress. These studies advanced the field by demonstrating actor–partner effects, highlighting how survivors’ and parents’ psychological well-being may be interrelated.

However, important limitations remain. In the study by Gutierrez-Colina and colleagues, the primary focus was on the association between individuals’ depressive symptoms and their own or their partners’ perceptions of the family environment, rather than on how perceptions of the family environment influenced depressive symptoms [19]. Similarly, Viola et al. examined associations among problem-solving skills, parent–adolescent cancer-related communication, dyadic functioning, and psychological distress among the adolescents with cancer [20]. Although this study incorporated a dyadic perspective, its assessment of the family environment was restricted to the parent–adolescent dyad and did not capture broader family-level environment factors. Most importantly, none of these studies examined the multidimensional symptom burden that encompasses both physical and psychosocial symptoms among AYAs affected by cancer.

To address these gaps, we conducted a secondary analysis using data collected from a previous study with Korean AYAs affected by cancer and their parents [21]. The original data were collected to answer the main research question regarding the association between parent–adolescent communication and use of constructive coping among Korean AYAs affected by cancer and the underlying mechanism. The parent study was informed by the Resilience in Illness Model, which emphasizes the individual and family perceived factors that support the development of AYAs’ resilience in the context of illness [22]. The family remains a central institution in Korean society, despite changes in family structure, including smaller household sizes and evolving family compositions [23], and the increasing influence of individualism [24]. The role of the family becomes particularly salient during the period of chronic illness [25]. A significant proportion of AYAs affected by cancer demonstrate a heightened reliance on parents for both psychosocial and financial stability throughout the continuum of cancer care [26,27]. Considering the critical role that the family plays, increasing knowledge related to the characteristics of a supportive family environment that contributes to alleviated symptom distress among AYAs affected by cancer is vital. This knowledge may highlight the importance of including the family in the care of AYAs affected by cancer. Thus, the overall purpose of this secondary analysis was to compare different perspectives on the family environment between AYAs affected by cancer and their caregivers and to examine which family environment variables (family cohesion and adaptability, perceived support from family, and family strength) were associated with symptom distress among AYAs affected by cancer. Selection of family environment variables was made based on available indicators of the family environment that were conceptually relevant to a supportive family environment contributing to individuals’ resilience. However, this subgroup analysis was not designed to test a comprehensive family-systems or dyadic theoretical model. The specific aims of this subgroup analysis were the following:

(1) Identify agreement between AYAs and their parents on perceptions of family cohesion, adaptability, social support, and family strength.

**Hypothesis 1:**  *AYAs and their parents would show moderate agreement across family cohesion, family adaptability, perceived family support, and family strength.*

(2) Examine whether AYAs and/or parents’ perceived family cohesion and adaptability, perceived social support from family, and family strength were associated with AYAs’ symptom distress.

**Hypothesis 2:**  *Higher levels of perceived family cohesion, family adaptability, family support, and family strength, as reported by both AYAs and parents, would be associated with lower symptom distress among AYAs.*

## 2. Materials and Methods

### 2.1. Design

This secondary analysis used data generated from a larger study that examined the relationship between parent–adolescent communication and the use of courageous coping among Korean AYAs with cancer, employing a cross-sectional, correlational design [21].

### 2.2. Setting and Population

The sample was accrued from a pediatric hematology-oncology outpatient clinic at a university-affiliated hospital in South Korea and via online recruitment efforts supported by the Korean Child Leukemia Foundation. For both settings, AYAs were eligible for inclusion if they (a) were aged between 11 and 26 years; (b) had been diagnosed with cancer at any stage, excluding those receiving end-of-life hospice care; (c) were aware of their cancer diagnosis; and (d) were able to communicate in Korean. Parents of eligible AYAs were also invited to participate. Exclusion criteria included cognitive impairments among AYAs or parents that would preclude informed consent, assessment or completion of the study surveys. AYAs were additionally excluded if they had a severely compromised immune status that would prevent participation in the study. Although the widely accepted definition of AYAs refers to adolescents and young adults aged between 15 and 39 years according to the National Cancer Institute [28], flexibility in working with sub-age groups according to the research question is required [29,30]. In general, there is no single age range for adolescence in Korea; the Youth Framework Act defines youth/adolescents (cheongsonyeon in Korean) broadly as individuals aged 9–24 years, with adolescence commonly understood as beginning around age 10 and emerging adulthood conceptualized as extending through the mid-20s [31]. Therefore, the age range was expanded to between 11 and 26 years, spanning early adolescence through emerging adulthood periods during which family relationships and support may remain especially relevant. A total of 144 Korean AYAs and 78 of their parents enrolled in the larger study. Detailed information on the original study is cited in previous publication [21]. This secondary analysis used data from matched AYA–caregiver dyads.

### 2.3. Measurements

#### 2.3.1. Sample Characteristics

Korean AYAs affected by cancer and their parents provided demographic information, including age, gender, and education, as well as clinical characteristics, including diagnosis, time since diagnosis, comorbidities, and current cancer treatment status. Parents of those recruited from the onsite clinic permitted research staff to access medical data to extract information about the AYAs’ diagnosis and recurrence status. In the online setting, parents confirmed the demographic or clinical characteristic information reported by AYA participants. The AYAs’ gender, age, and current cancer treatment status, as well as the parents’ age and education status, were included as possible covariates when testing the hypothesized associations.

#### 2.3.2. Family Cohesion and Adaptability (FACA)

Both Korean AYAs with cancer and their parents reported their perceptions regarding FACA by completing the 30-item Family Adaptability/Cohesion Scale (Faces II) [32], which was translated into Korean by Lee et al. [33]. The FACA scale assesses how family members interact, make decisions, and manage change, as well as how close or connected they feel to one another. Items were rated on a 5-point rating scale from 1 (almost never) to 5 (almost always). Exemplar items include “family members are supportive of each other during difficult times” and “our family tries new ways of dealing with problems”. A higher score indicates a greater level of family cohesion. The reliability and validity of the measurement were confirmed among AYAs with cancer aged 10–26 years in the United States (α = 0.90 (cohesion), α = 0.85 (adaptability), α = 0.92 (total scale) [29]. The validity and reliability were confirmed among Koreans (α = 0.70) [33]. In the current study, Cronbach’s alpha of the total FACA scale was 0.935 among AYAs with cancer and 0.925 among parents of AYAs with cancer.

#### 2.3.3. Family Strength (FS)

Both Korean AYAs with cancer and their parents reported their perceptions regarding family strength by completing the 12-item Family Strength Scale [34]. The scale measures how family members perceive the family as a worthy group (pride) and their sense of mastery of family (accord). Items were rated on a 5-point rating scale from 1 (strongly disagree) to 5 (strongly agree). Exemplar items include “we can express our feelings” and “family members feel loyal to the family”. A higher score indicates a greater level of family strength. The reliability (Cronbach’s alpha of 0.90) and validity were confirmed among AYAs with cancer aged 10–26 years in the United States. In this study, the Family Strength Scale was translated into Korean, and the Cronbach’s alpha was 0.910 among Korean AYAs and 0.909 among their parents.

#### 2.3.4. Perceived Social Support from Family (PSS-Fa)

Both Korean AYAs with cancer and their parents reported their perceptions regarding family support by completing the 20-item PSS-Fa scale [35]. The PSS-Fa was translated into Korean by our research team [36]. Items were rated on a 5-point rating scale from 1 (totally disagree) to 5 (totally agree). Exemplar items include “the family gives me the moral support I need” and “I rely on my family for emotional support”. A higher score indicates a greater level of family support. The reliability and validity of the measurement were confirmed among AYAs with cancer aged 11–26 years in the United States (Cronbach’s alpha of 0.93) [29]. The Cronbach’s alpha in this study was 0.911 among Korean AYAs with cancer and 0.928 among parents of AYAs with cancer [36].

#### 2.3.5. AYA’s Symptom Distress (SDS)

AYAs-reported symptom distress was assessed using a modified Korean-translated version of the McCorkle Symptom Distress Scale (SDS) [37], with the final analytic score calculated from 10 retained items. The translation of the original SDS was performed by three Korean researchers with expertise in nursing and social science, all of whom were fluent in English, using a forward–backward translation. Throughout the translation process, the research team continuously evaluated semantic accuracy through iterative discussions with a native-English-speaking nursing researcher with expertise in AYAs and symptom distress to ensure the fidelity of meaning. The original SDS has 13 items assessing patients’ self-reported symptoms in the domains of nausea, appetite, insomnia, pain, fatigue, bowel function, concentration, appearance, breathing, outlook, and cough. In this study, a symptom distress total score was calculated by summing the 10 items only. Two items, regarding outlook and cough, were inadvertently removed from the translation by researchers, and an additional item related to breath difficulty was removed due to low factor loading of 0.13 in the principal component analysis (PCA). The scale uses a 5-point Likert scale (1 = no problem to 5 = maximum amount of problem). The possible score ranges from 10 to 50, with higher scores indicating greater distress. The Cronbach’s alpha of the original SDS ranged from 0.75 to 0.83. The reliability and validity were confirmed with Korean AYAs in this study. Cronbach’s alpha for the total score used in this study was 0.87. Although the final 10-item scale demonstrated strong internal consistency in this sample, it should be interpreted as a study-specific symptom distress index rather than a validated shortened SDS. Future studies should administer the full original SDS or use a validated symptom distress measure appropriate for the target population.

### 2.4. Data Collection

The study was conducted in two settings from 25 June 2019, to 31 August 2020. First, in-person recruitment and data collection were conducted at a university-affiliated hospital in Seoul, Korea. Healthcare providers at the clinical site referred eligible participants to the research team. After informed written and verbal consent was obtained, AYAs and their parents were asked to complete a separate booklet of questionnaires that included the self-report measures used in this analysis. Throughout the process, research assistants provided instructions and answered any questions. During data collection, AYAs and their parents were explicitly reminded to complete the questionnaires with reference to their shared family system, ensuring that responses reflected perceptions of the same overall family environment. This approach was intended to enhance conceptual alignment between respondent perspectives and reduce measurement inconsistency in analysis. When participants felt tired, they took a rest or completed the survey at home and returned it at their next clinic visit. Most AYA participants completed the survey while receiving intravenous fluid infusion or waiting for their physician. For those recruited online through the Korean Leukemia Foundation, once researchers received the online consent, they contacted the potential participants to confirm their consent and eligibility. With eligibility confirmed, researchers sent a survey link to the participants to complete via Survey Monkey, which provided secure database storage and complied with HIPAA regulations. The survey took about 40 min to complete in both settings.

### 2.5. Ethical Approval

The original study was conducted upon the IRB approval at both Korean study sites and North American site for data transfer and analysis. Additional IRB was waived for this subgroup analysis as de-identified data were used.

### 2.6. Data Analysis

All collected data were analyzed by SPSS 29 software for Windows (IBM Corp, Armonk, NY, USA). The demographic and clinical characteristics of both AYAs and their parents are presented as frequencies and percentages for categorical variables or means and standard deviations for continuous variables. The scores of all variables, including FACA, FS, PSS-FA by both AYAs affected by cancer and their parents, and AYAs’ SDS, are presented as means and standard deviations. The degree of agreement between AYAs and their parents on perceptions of those variables was assessed with the intra-class correlation coefficient (ICC), a two-way random effects model. Additionally, paired t-tests were performed to evaluate the extent of mean differences between AYAs and parents regarding their perceptions of FS, PSS-Fa, and FACA.

Multiple regression analyses were employed to examine whether AYAs and/or parent-reported variables (FACA, PSS-Fa, FS) were associated with AYAs’ SDS. As the preliminary step, Pearson correlations were run to analyze the relationship between the independent variables (AYA and parent perceived FACA, FS, PSS-Fa) and covariates (treatment status, AYAs’ age, gender, parent’s age, and education level), with the dependent variable of AYAs’ SDS. Initially, a full model was tested with all variables that were significantly correlated with AYAs’ SDS. Because of high multicollinearity among the family environment variables, the stepwise regression analysis was used as an exploratory variable-selection approach to identify a parsimonious model of variables most strongly associated with the AYAs’ symptom distress. Finally, the interaction among the significant key variables was tested using all AYA data regardless of whether they had matched parental data. All statistical tests were 2-sided, and *p* < 0.05 was considered statistically significant.

## 3. Results

### 3.1. AYA Demographic and Clinical Characteristics

Self-reported data from a total of 113 AYAs, 56 (49.6%) of whom had matched parental data, were used in this secondary analysis. Table 1 details the demographic and clinical characteristics of the 113 AYAs in this sample. The mean age was 16.6 (SD: 3.8), with 30.4% in high school and 29.7% attending a college/university. The sample was 59.3% female and 40.7% male. In terms of cancer diagnosis, most had a solid tumor cancer (45.1%) and 15.9% reported a cancer recurrence, and 42.5% were currently receiving cancer treatment. At the time of the survey, 15.9% of Korean AYAs with cancer in this study did not live with their fathers, and 2.7% of them did not live with their mothers. The only significant differences between AYAs recruited through clinical sites versus online were education level (*p* = 0.006), with a higher proportion of participants reporting a middle school education in the clinical-site group, and comorbidity status (*p* = 0.001), with more AYAs recruited from clinical sites reporting comorbidities. Symptom distress did not differ significantly by recruitment setting.

Table 2 presents the demographic characteristics of 56 parents. The mean age of the parent was 46.4 (SD: 5.87). Most were mothers (89.3%), and more than half of the parent participants were not currently employed or working. The majority of the parent participants reported their economic status as medium ($2578–$5757 per month, 88.9%). More than half of parents completed either a college-level education or graduate/professional schools.

### 3.2. Primary Correlations Between the Family Environment Variables and AYA Symptom Distress

Correlations were examined between AYAs’ symptom distress (dependent variable) and other family environment variables (parent and AYAs’ perceived family adaptability and cohesion, family support, and strength) and covariates (AYAs’ age, gender, treatment status, and parents’ age and education level). The five covariates included in the models were selected based on their clinical relevance and suitability for the available sample size. Although other factors, such as cancer type or treatment modality, may also be clinically important, they were not included because the small number of participants within each category could have produced unstable estimates. These correlations indicated that all family environment variables from both Korean AYAs and their parents’ views were significantly associated with the AYAs’ SDS (*p* < 0.05), indicating that both parents and AYAs perception of their family environment may play an important role in AYAs’ symptom distress (Table 3). The correlations between the family environment variables and AYAs’ symptom distress were all negative and ranged from low to moderate correlations (r = −0.243 to −0.523). Furthermore, AYAs’ symptom distress was highly correlated with AYAs’ reported FS (r = −0.523, *p* < 0.001). Among the five covariates, treatment status was the only one significantly correlated with AYAs’ symptom distress (*p* = 0.006). Parents’ age, educational level, AYAs’ gender, and age were not correlated with AYAs’ symptom distress (*p* > 0.05).

### 3.3. Agreement Between AYAs’ and Their Parents’ Perceived Family Environment

To determine the extent of agreement between AYAs’ and parents’ perceptions regarding family cohesion and adaptability, family support, and family strength, ICCs were estimated. AYAs and their parents showed their highest agreement on perceptions regarding family cohesion and adaptability (ICC = 0.612, *p* < 0.001), and the lowest agreement on perceptions about family support (ICC = 0.374, *p* < 0.001). Means and standard deviations of AYA and parent responses are displayed in Table 3. AYAs reported significantly lower perceived family support than their parents, with a mean difference of 3.35 points (AYA M = 43.54, SD = 8.70, parent M = 46.89, SD = 8.01; *p* < 0.001, d = 0.48). This difference represented a small to moderate effect. However, AYAs and their parents had no significant difference in how cohesive and adaptable (*p* = 0.086, d = 0.21) they perceive their families to be, and their family strength. (*p* = 0.240, d = 0.14).

### 3.4. Factors Associated with AYAs’ Symptom Distress

Initially, a full regression model was run, including all variables with treatment status and AYA’s age as the only covariates, using data from 54 AYA–parent dyads who had complete data regarding the key family environment variables. However, this suggested evidence of potentially problematic (moderate) multicollinearity, with VIF values ranging from 1.11 to 5.37. The highest VIF was for parent perceived family strength (VIF = 5.37), which meets the commonly used criterion of VIF ≥ 5 as indicative of moderate multicollinearity [38,39]. Therefore, a stepwise regression as an exploratory variable-selection approach to identify a more parsimonious model, while reducing redundancy among variables, was then run (Table 4). Results indicated that the only meaningful variable associated with AYAs’ symptom distress was AYA-perceived family strength (F = 10.234, *p* < 0.001, R^2^ = 0.380, adjusted R^2^ = 0.343), including treatment status and age as covariates. A total of 38% of the variance in AYAs’ symptom distress was explained by AYAs’ report of family strength and treatment status, and age.

The interaction between family strength (AYA) and treatment status was tested. Because the regression model did not include any parent perceptions, a second regression model was run using 111 AYAs who had complete data on treatment status and symptom distress score, regardless of whether they had matched parental data. The interaction was significant using the larger sample and increased power (n = 111, *p* < 0.001). The results are presented in Table 5. When off treatment (ts = 0), for every unit increase in AYA reports of family strength, AYA perceived symptom distress decreased by 0.28; and when on treatment (ts = 1), for every unit increase in AYA reports of family strength, the AYAs’ symptom distress decreased by 0.59. Therefore, the effect of AYAs’ perceived family strength on symptom distress is stronger when AYAs are on treatment.

## 4. Discussion

This study sought to describe how Korean AYAs affected by cancer and their parents perceive their family environment in terms of family cohesion and adaptability, family strength, and perceived support from family, with a focus on the extent of agreement between AYA and parent reports. An additional aim was to identify the family environment factors associated with Korean AYAs’ multidimensional symptom distress, including both physical and psychological symptoms. Korean AYAs affected by cancer and their parents showed moderate agreement in their perceptions of family strength and cohesion and adaptability, but poor agreement in their perceptions of family support. Agreement reflects similarity or directions in their views between AYA and caregiver reports, not whether perceptions are positive or negative. High agreement can occur even when both parties rate family functioning as low, whereas low agreement indicates discordant perceptions. For example, in this study, Korean AYAs perceived their family strength as significantly lower than did their caregivers, although they had moderate agreement in their perception of family strength. This pattern is consistent with prior findings among American AYAs affected by cancer, which have shown that AYAs tend to evaluate their family environment more negatively than their caregivers. However, to our knowledge, no prior studies have examined these perceptions among Korean AYAs, limiting direct cross-cultural comparisons.

Another important finding is the low agreement between Korean AYAs affected by cancer and their parents in the perception of their family support. In this study, the measurement of PSS-FA measures individuals’ perceptions of support received from family members; thus, parents’ report may reflect support drawn from multiple family relationships (e.g., spouse or other family members), rather than support provided specifically by the AYAs. Accordingly, the observed low agreement may indicate differing support contexts and needs within the family system, rather than a lack of support.

The divergent views on the family environment, represented by low to moderate agreement in the perceptions about their family environment, suggest that the family environment is not experienced uniformly within the family system. Although AYAs and parents share the same family context, they may differ in how they perceive the family cohesion, adaptability, family strength, and perceived support. These discrepancies may originate from the dramatic developmental changes that AYAs experience [40,41,42,43], caregiving roles and expectations for autonomy, and emotional responses to cancer experience. Particularly, early adolescence is a transition period characterized by an increased need for independence and autonomy. However, AYAs and parents often have different expectations regarding the appropriate timing of transitions in authority, autonomy, and responsibilities [43]. AYAs want changes in parent–AYA relationships, often causing conflicts between them due to different perceptions. AYAs may perceive their family as weak or not supportive when the expected changes do not occur as quickly as they desire. Thus, the modest level of disagreement observed in this study highlights the importance of assessing both AYA and parent perspectives in clinical practice, rather than relying on a single family member’s report.

Clinically, these findings suggest that healthcare providers should consider assessing both perspectives regarding the family environment when supporting AYAs affected by cancer and their parents. In Korean cultural context, parents often play a highly involved caregiving role and may serve as key patient advocates in care-related decision making [44], which may inadvertently limit opportunities for AYAs to express their own thoughts, preferences, and concerns. This pattern may be influenced by familism, filial piety, and Confucianism, emphasizing family interdependence, parental responsibility, respect for elders, and family harmony [45]. Because maintaining harmony and protecting family members from emotional burden are often highly valued, AYAs and parents may be less likely to openly express differing views about family relationships and support. Thus, assessing both perspectives regarding the family environment may help clinicians identify potential areas of miscommunication, unmet support needs, differences in autonomy expectations, or mismatched perceptions of family cohesion, adaptability, family strength, and support. Routine family-centered screening and conversations that invite both AYAs’ and parents’ perspectives may support more culturally responsive care.

Family cohesion and adaptability, perceived support from family, and family strength from both AYAs’ and their parent perspectives were examined to determine whether they were associated with AYAs’ symptom distress. Initially, it was hypothesized that both parents’ and AYAs’ views regarding their family environment would play an important role in determining AYAs’ symptom distress. Prior research has consistently supported the importance of family cohesion, adaptability, and perceived support in the cancer context [12,46,47,48]. However, contrary to this evidence, AYAs’ self-reported family strength emerged as the only significant—and the strongest—factor associated with AYAs’ symptom distress. Family strength is conceptually and empirically intertwined with other dimensions of the family environment, including family cohesion, adaptability, and perceived family support, all of which contribute to the broader family context [12]. However, family strength may capture a more integrated and proximal appraisal of the family system, reflecting not only the presence of supportive relationships but also a deeper sense of family identity, competence, shared purpose, and collective efficacy in managing cancer-related changes. In this sense, perceived family strength may overlap conceptually with family resilience and coping, as it reflects AYAs’ perceptions that their family can work together, mobilize resources, and maintain a sense of stability during challenging experience. Thus, the finding that only the AYAs’ perceived family strength was significantly associated with symptom distress does not necessarily suggest that cohesion, adaptability, or support are unimportant. Rather, family strength may represent a more comprehensive and clinically salient construct through which broader family processes are translated into AYAs’ symptom experiences. Additionally, the present findings extend existing knowledge by highlighting the critical role of family strength in alleviating symptom distress, particularly during periods of intensive cancer treatment, given that AYAs under treatment, compared to those who were off-treatment, reported up to four times greater relief in symptom distress associated with enhanced family strength. Symptom distress during intensive treatment is both heightened and persistent among AYAs affected by cancer [5]; therefore, fostering perceptions of family strength—characterized by mutual valuing among family members and a shared sense of competence and mastery in the family environment—may be especially important for this population.

The results of nonsignificance among other family environment variables, such as family cohesion and adaptability and perceived support from family, appear inconsistent with previous findings, which have emphasized family cohesion and adaptability as core values to the positive family environment in the cancer context [21,46,47,48,49], and even decreased symptom distress among the adult cancer population undergoing hematopoietic stem cell transplantation [50]. A possible explanation for the unexpected outcomes related to family cohesion and adaptability may lie in the multicollinearity between family cohesion and adaptability, and family strength. As noted above, these variables shared substantial overlapping variances; thus, the effect of family adaptability and cohesion may have been attenuated after family strength was included in the model. Another potential explanation may be related to the Korean cultural context. Traditionally, Korean society has emphasized the importance of family cohesion and bonding, as well as a hierarchy within the family structure [24]. Despite the benefits of family cohesion to family functioning, there have been warnings about the potential drawbacks of excessive emphasis on family bonding in Korean culture, particularly regarding children’s normal development [25,51]. Another possible explanation might be related to AYAs’ willingness to protect their parents from knowing about their suffering. In our previous study with Korean AYAs with cancer, we found that AYAs struggle to share their concerns and sufferings due to their eagerness to protect their family members from knowing the negative aspects of their cancer journey [52]. However, existing evidence supports that suppression of emotions ultimately negatively affects the alleviation of symptom distress [53,54].

Moreover, neither caregivers’ nor AYAs’ perceived family support was associated with AYAs’ symptom distress. This finding suggests that global perceptions of family support may not directly translate into symptom-level outcomes, which are often shaped by more proximal biological, treatment-related, and psychological factors [55,56]. In addition, the observed divergence of perception of family support between AYAs and their parents may limit the extent to which family support functions as effective buffer against AYAs’ symptom distress.

To our best knowledge, this is the first study to examine whether parents’ perception of their family environment was associated with AYAs’ symptom distress. However, we found that none of the parent-reported variables were associated with AYAs’ symptom distress once AYAs’ perception of family strength was included. The inclusion of AYAs’ perception only could reflect that AYAs’ own perceptions may be more proximally related to their own symptom distress than parents’ perceptions, because symptom distress is a subjective experience reported by AYAs themselves. Thus, AYAs’ appraisal of the family environment may have a more direct association with how they experience and report physical and psychological symptom distress. However, given the parental influence on their AYAs affected by cancer [54,55], this finding was unexpected. As noted above, multicollinearity among the family environment variables from both parent and AYAs’ perspectives might explain this. Particularly, parents’ perceptions of their family strength were excluded in the regression model due to multicollinearity. Thus, exclusion of the parents’ perceived family environment variables should not be interpreted as evidence that parent variables lack importance, because parents’ perceptions remain theoretically and clinically important. In the previous literature, the impact of parents’ psychological health status, such as parents’ distress [57], depression [58], and communication [59], on AYAs’ adjustment, distress, and quality of life has been widely explored. This inconsistent finding warrants further investigation.

### 4.1. Implications for Nursing Research and Practice

These findings have important clinical implications for healthcare providers. Given that AYAs’ perceived family strength emerged as the most salient factor associated with symptom distress, healthcare providers should prioritize AYAs’ subjective appraisals of the family environment during clinical assessment, rather than relying solely on parent reports.

Secondly, clinicians should recognize that family strength reflects an integrative appraisal on multiple interrelated family processes, including cohesion, adaptability, and support. Clinical assessments that consider these dimensions collectively may better capture families’ adaptive capacity than single-domain screening.

Third, discrepancies in perceived family support between AYAs and their parents may signal divergent support needs or experiences within the family system. Creating safe opportunities for AYAs and their parents to independently express their needs and identify unmet support may be important for effective symptom management and psychosocial care planning. Importantly, caregiver burden and psychological well-being are also recognized as key factors in family adjustment among AYAs affected by cancer [19,58]. Thus, further research is needed to examine how insufficient perceived support among parents and AYAs affected by cancer may indirectly affect symptom distress.

Recently, the importance of the supportive family environment has been increasingly recognized. Several research organizations, such as National Cancer Institute (NCI) and Oncology Nursing Society (ONS), have prioritized research focusing on the impact of familial, socioeconomic, and other environmental factors on survivor outcomes and development of targeted interventions to reduce the burden of cancer for AYA survivors [60,61]. Based on findings of this study, which indicate the lack of direct association between the family environment factors and AYAs’ symptom distress, future studies should examine the indirect pathway through which the family environment variables influence symptoms, such as coping strategies, emotional regulation, sleep or treatment adherence [58]. Additionally, further future studies are required that focus on the broad family environment. In this study, we examined the subjective psychological family environment, which refers to the emotional and social climate within a family, encompassing how individuals perceive and experience family dynamics; however, future studies examining both the psychological and the physical family environment, such as family income, living space, and so on, are recommended. A future longitudinal design would be helpful in clarifying how the family strength and related family processes evolve and how their influences on symptom distress may vary across the cancer trajectory. Finally, as one of the first studies to examine the family environment perceptions among Korean AYAs affected by cancer, future research should explore how cultural norms regarding family roles, interdependence, and emotional expression shape both agreement and discrepancies in the perceived family environment across diverse populations. Qualitative or mixed-methods studies that directly capture the lived experiences of AYAs and their families would provide a richer understanding of how the family environment is experienced, interpreted, and negotiated in the context of cancer, beyond what can be explained through quantitative scores alone.

### 4.2. Strengths and Limitations

To our knowledge, a key strength of this study is that it is the first to compare the parent and AYAs’ perspectives on the family environment in the context of Korean AYA cancer. This study contributes new knowledge by identifying whose perspective on specific dimensions of the family environment is most relevant to the symptom distress. Importantly, it extends beyond prior work, which has been largely limited to psychological health outcomes, by demonstrating the influence of the family environment on both physical and psychological symptom distress. The findings of this study should be interpreted in light of the following limitations. Overlaps were found among the family environment variables in this study, which caused multicollinearity issues. Due to the nature of the family environment construct, it is not possible to choose completely separate variables. Another limitation of this study is the inclusion of a broad age range from 11 to 26 years, spanning early adolescence to young adulthood. Although age was included as a covariate in the regression models and was not statistically significant, the sample size may have limited our ability to detect age-related differences. Given developmental differences in autonomy, parent–child relationships, decision making, and reliance on family support [62], the inclusion of younger adolescents aged 11–14 may limit the generalizability of the findings to studies using more traditional AYA age definitions. It is also important to acknowledge that the majority of parent participants in this study were mothers, which limited our ability to capture potentially different perspectives from fathers. Finally, as this study was a secondary analysis, the selection of variables was constrained by the indicators available in the parent dataset rather than being guided by theoretical framework. Consequently, parent–adolescent communication from both AYAs with cancer and their parents could not be included because incomplete responses to the communication measures would have substantially reduced analytic sample size. Future studies should prospectively examine the family environment variables within a stronger theory-driven dyadic framework and include the parent–adolescent communication, given its role as a core family function [17,63,64]. Finally, the findings cannot be generalizable to AYAs affected by cancer and their families from different cultural backgrounds.

Based on these limitations, we recommend that future studies include carefully selected family environment variables based on a strong theoretical framework. Specifically, for assessing the perceived support, future research should move beyond global, individual-level measures of the family support to incorporate dyadic or family-level assessments that distinguish support provided, received and perceived by different family members. Additionally, future studies with larger samples, including more fathers, would allow further examination of whether the association between family environment and symptom distress differs across developmental subgroups.

## 5. Conclusions

In summary, this secondary analysis study provides insight into AYAs affected by cancer and their symptom distress. AYAs’ perceived family strength emerged as the only family environment factor that remained significantly associated with AYAs’ symptom distress in the final model, suggesting that AYAs’ own appraisals of family strength may be particularly relevant to their symptom experience. Healthcare providers caring for Korean AYAs need to assess AYAs’ perception of their families’ pride and sense of mastery and encourage them to be involved across the cancer trajectory.

## Figures and Tables

**Table 1:** AYA characteristics (N = 113).

Characteristics	Statistic
Age, in years (n = 113)	16.6 ± 3.8
Female gender	67 (59.3%)
Religious (n = 112)	61 (%)
Educational level (n = 111)	
Elementary school	29 (13.8%)
Middle school	28 (26.1%)
High school	36 (30.4%)
College or university	18 (29.7%)
Time since diagnosis (n = 113)	
Less than 1 year	27 (23.9%)
Between 1 and 2 years	15 (13.3%)
Between 2 and 3 years	18 (15.9%)
Between 4 and 6 years	34 (30.1%)
Over 6 years	19 (16.8%)
Cancer diagnosis (n = 109)	
Hematologic malignancy	43 (38.1%)
Solid tumor	51 (45.1%)
Brain tumor	12 (10.6%)
Not specified	2 (1.8%)
Rare cancer	1 (0.9%)
Ever relapsed cancer diagnosis (n = 112)	18 (15.9%)
Currently receiving cancer treatment (n = 113)	48 (42.5%)
One or more comorbidities (n = 90)	34 (30.1%)

Note: AYA = Adolescent and young adults; n (%) reported with exception of mean ± standard deviation (SD) for age.

**Table 2:** Parents’ characteristics (N = 56).

Characteristics	n (%)
Age, in years (n = 56)	46.4 ± 5.87
Relationship to AYA	
Mother figure	50 (89.3%)
Father figure	6 (10.7%)
Monthly economic status	
High	2 (3.7%)
Medium	48 (88.9%)
Low	4 (7.4%)
Current employment status	
Employed/working	25 (44.6%)
Not employed/not working	31 (55.4%)
Parent educational level	
Middle school graduate	3 (5.4%)
High school graduate	23 (41.1%)
College level graduate	25 (44.6%)
Professional or graduate level	5 (8.9%)
Religious	39 (69.6%)

Note: n (%) reported with exception of mean ± standard deviation (SD) for age; High economic status defined as $5758 or greater per month, medium as $2578–$5757, and low as $2577 or less. Income categories were originally reported in Korean won and converted to U.S. dollars.

**Table 3:** AYA and parent self-report responses.

Measures	n	Mean ± SD (Min, Max)	Number of Items (Possible Range)	Pearson Correlation Coefficient (*r*) ^a^	Standardized Cronbach’s Alpha
AYA report of SDS	113	17.7 ± 6.5 (10, 50)	10		0.87
AYA report of FCA	111	107.9 ± 17.5 (58, 144)	30	−0.424 **	0.94
AYA report of FS	112	42.6 ± 8.5 (19, 59)	12	−0.523 **	0.91
AYA report of PSS-F	113	77.2 ± 11.9 (39, 99)	20	−0.331 **	0.91
Parents report of FCA	57	112 ± 14.4 (68, 138)	30	−0.243 *	0.92
Parents report of FS	57	46.7 ± 8.1 (17, 60)	12	−0.313 **	0.91
Parents report of PSS-F	56	79.8 ± 11.6 (33, 96)	20	−0.305 **	0.93

Note: ^a^ Reflects the correlation with symptom distress as a dependent variable; * *p* < 0.05, ** *p* < 0.001; SDS = symptom distress; FCA = Family cohesion and adaptability; FS = Family strength; PSS-F = perceives social support from family.

**Table 4:** Summary of stepwise regression: factors associated with AYA symptom distress (N = 54).

	Variable	B	SE B	β	VIF
Model 1 (Full Model)	Treatment Status	4.051 **	1.293	0.370	1.107
	Age	0.239	0.199	0.144	1.139
	AYA-FCA	−0.104	0.070	−0.340	4.163
	AYA-FS	−0.239	0.133	−0.390	3.763
	AYA-PSS-F	0.091	0.086	0.212	3.153
	Parent-FCA	0.098	0.095	0.260	5.074
	Parent-FS	0.037	0.175	0.055	5.366
	Parent-PSS-F	−0.104	0.115	−0.223	4.851
	*R*^2^	0.434			
	*F*	4.307 ***			
Model 2 (Reduced Model)	Treatment Status	3.665 **	1.226	0.335	1.011
	Age	0.243	0.186	0.146	1.016
	AYA-FS	−0.281 ***	0.068	−0.460	1.009
	*R*^2^	0.380			
	*F*	10.234 ***			

Note: AYA = Adolescent and young adult; FCA = Family cohesion and adaptability; FS = Family strength; PSS-F = perceives social support from family; *R*^2^ = coefficient of determination, representing the proportion of variance in the outcome variable; F = F-Ration; B = Unstandardized Regression Coefficient; SE B = Standard Error of B; β = standardized regression coefficient; VIF = Variance Inflation Factor; VIF < 5 is acceptable, VIF 5–10 represents moderate concern, VIF > 10 represents serious multicollinearity problem ** *p* < 0.01, *** *p* < 0.001.

**Table 5:** Summary of stepwise regression: factors associated with AYA Symptom distress, including the interactions between treatment status and AYAs’ FS (N = 111).

Variable	B	SE B	β
Treatment status	23.992 ***	4.901	1.841
AYA-age	0.399 **	0.125	0.231
AYA-FS	−0.149 *	0.067	−0.197
TX x AYA-FS	−0.439 ***	0.114	−1.444
*R*^2^	0.457		
*F*	22.488 ***		

Note: AYA = Adolescent and young adult; FCA = Family cohesion and adaptability; FS = Family strength; PSS-F = perceives social support from family. *R*^2^ = coefficient of determination, representing the proportion of variance in the outcome variable; F = F-Ration; B = Unstandardized Regression Coefficient; SE B = Standard Error of B; β = standardized regression coefficient; VIF = Variance Inflation Factor; VIF < 5 is acceptable, VIF 5–10 represents moderate concern, VIF > 10 represents serious multicollinearity problem * *p* < 0.05, ** *p* < 0.01. *** *p* < 0.001.

## Data Availability

The data that support the findings of this study are available from the corresponding author, H.Y.S., upon reasonable request.

## Abbreviations

The following abbreviations are used in this manuscript:

AYA	Adolescent and young adults
FACA	Family cohesion and adaptability
PSS-FA	Perceived social support from family
FS	Family strength
SDS	Symptom distress
